# Supplementary material for: 4′‐Phosphopantetheine corrects CoA, iron, and dopamine metabolic defects in mammalian models of PKAN
Source: EMBO Mol Med. 2019 Oct 29;11(12):e10489. doi: 10.15252/emmm.201910489 (PMC6895607; doi:10.15252/emmm.201910489)
Supplement: Supplementary file 1 — Appendix [file EMMM-11-e10489-s001.pdf]

Appendix Table of Contents

- Appendix Table S1; QPCR primer sequences p1
- Appendix Table S2; P values p2
- Appendix Figure S1 p4
- Appendix Figure S2 p5
- Appendix Figure S3 p6
- Appendix Figure S4 p7
- Appendix Figure S5 p8
- Appendix Figure S6 p9

Appendix Table S1

QPCR Primers

Species	Target	Forward	Reverse
Human	*18s*	AGTCCCTGCCCTTTGTACACA	GATCCGAGGGCCTCACTAAAC
Human	*COASY*	GCTGAAGATACTCACGGACATT	CAATCACACACACACGCTTTC
Human	*Mt-ATP6*	ThermoFisher Hs02596862_g1	
Human	*Mt-CO1*	ThermoFisher Hs02596864_g1	
Human	*Mt-CYB*	ThermoFisher Hs02596867_s1	
Human	*Mt-ND1*	ThermoFisher Hs02596873_s1	
Human	*PPCDC*	CAACTGAGAGAGCCAAACATTTC	GGCTCTTCCATATCTCCCATTC
Human	*PPCS*	CCTGTCTCTGAAATGCCTGAA	TGGGAGCCCAATCTTTAACC
Human	*TFRC*	GACAATGCTGCTTTCCCTTTC	TCCATGGTGGTACCCAAATAAG
Mouse	*Calb1*	AAGGCTGGATTGGAGCTATC	GACGTGAGCCAACTCTACAA
Mouse	*Coasy*	GGAGGCCTTTGGAACAGATATT	GAGGATCTTCATCTGCTTCTTGT
Mouse	*Cpt1c*	GTTTCCTCTGGAGGTGGATTT	GCTGGAGATATGGAAGGTGATT
Mouse	*Drd1*	TCCCAGATCGGGCATTTG	CTCTTCCTGGTCAATCTCAGTC
Mouse	*Drd2*	GCCATTGTTCTTGGTGTGTTC	TAGAGGACTGGTGGGATGTT
Mouse	*Gabra3*	ATCCGGTCTAGTACAGGAGAAT	GATACATGGCAAGTAGGTCTGG
Mouse	*Gabra6*	GAGTCAGTCCCAGCAAGAAC	GCATAGGACACTTTGGGTAGAG
Mouse	*Gapdh*	TGCACCACCAACTGCTTAGC	GGCATGGACTGTGGTCATGAG
Mouse	*Hepc*	TTGCGATACCAATGCAGAAG	TGCAACAGATACCACACTGG
Mouse	*Ireb2*	GACAAGCACTGGAAAAGAGTTC	ACGAGTCATGAAAGATACCACG
Mouse	*Pank1*α	GTTCGCCCAGCATGATTCTC	CTTAACCAGGGTTCCACCGAT
Mouse	*Pank1*β	CTGAGCCTAACTCCATTCAACT	TCCACCGATATCCATACCAAAC
Mouse	Pan-*Pank1*	GGACATTTACGGAGGAGACTATG	CTTTGCTGATGGACTCTCTCTT
Mouse	*Pank3*	TCACTGGGAACCAAAGGATAAA	CAGTGAGGGATACTTCCCATTATAG
Mouse	*Ppcdc*	TAACAACAGAGAGAGCCAAACA	GCTTCCACATCTCCCATTCA
Mouse	*Ppcs*	CTCTCAGTCCATTAGGCTCTTC	GGATCTTGTGTTCAGGCATTTC
Mouse	*Tfrc*	GAGTATCACTTCCTGTCGCCCTATG	GCTGAGAGAGTGTGAGAGCCAGAGC
Mouse	*Th*	CCCTACCAAGATCAAACCTACC	CTGGATACGAGAGGCATAGTTC

Appendix Table S1 QPCR primers are listed for each target gene

Exact P Values and Types of ANOVA Analyses

Figure		P				ANOVA
Fig 1C		*Ppcs*	*Ppcdc*	*Coasy*		
	GP	3.2000E-03	8.4463E-01	9.3703E-07		1w
	SN	1.5323E-01	1.5681E-01	5.0979E-02		1w
	Cerebellum	2.6651E-01	3.5626E-01	8.6756E-01		1w
Fig 1D	Within vehicle	7.6512E-03				2w
	Within KO	9.9097E-05				
Fig 2A		*Tfrc*	*Ireb2*	*Hamp*		
	GP	2.2904E-05	2.7871E-02	6.8690E-03		1w
	SN	5.8679E-01	3.7452E-01	1.4969E-01		1w
	Cerebellum	4.1119E-02	2.9212E-01	5.1926E-02		1w
Fig 2B	Cytosol	3.4071E-02				1w
	Mitochondria	3.5259E-02				1w
Fig 2C		Mitochondrial	Cytosol			
	Brain	1.4553E-04	3.8716E-01			1w
	Liver	4.6571E-01	5.0120E-01			1w
Fig 2D	Within vehicle	2.8158E-03				2w
	Within KO	2.1842E-03				
Fig 2E	Within vehicle	1.8000E-02				2w
	Within KO	2.0000E-02				
Fig 2F		*Tfr1*	*PDHE1a1*			
	Within vehicle	3.1626E-06	1.3863E-01			2w
	Within KO	7.8443E-04	5.1421E-02			
Fig 3A		*Drd1*				
	GP	5.3339E-06				1w
	SN	4.3633E-01				1w
	Cerebellum	7.3619E-01				1w
		Drd1				
	Within vehicle	1.3818E-04				2w
	Within KO	1.2694E-05				
Fig 3B-C		*Drd2*	*Gabra3*			
	GP	2.7109E-02	9.2253E-01			1w
	SN	9.9616E-01	5.6443E-01			1w
	Cerebellum	9.3350E-01	2.1192E-01			1w
Fig 3D	WT vs. KO	1.4058E-02				1w
Fig 4A		*COASY*				
	Fibroblasts	8.6730E-05				1w
	Lymphoblasts	4.5552E-03				1w
Fig 4B		*COASY / Coasy*				
	Human	2.8232E-08				1w
	Mouse	4.7685E-03				1w
Fig 4C	OCR	1.7873E-03				1w
	ECAR	3.0106E-03				1w

Con't

Exact P Values and Types of ANOVA Analyses

Figure		P				ANOVA
Fig 4D						
	Within vehicle	1.3744E-02				2w
	Within KO	1.5759E-02				
Fig 4E	*MT-ND1*	4.8595E-02				1w
	MT-CYB	4.8953E-02				1w
	MT-CO1	4.7521E-02				1w
	MT-ATP6	4.9782E-02				1w
Fig 4F	Ctl vs. PKAN	3.6231E-02				1w
Fig 5A		*Pank1a*				
	GP	5.0287E-03				1w
	SN	8.8661E-01				1w
	Cerebellum	5.3904E-01				1w
	Pank1a	5.0287E-03				1w
	Pank1b	6.9087E-01				1w
	Total	1.7742E-02				1w
Fig 5B		*Pank3*				
	GP	7.1749E-01				1w
	SN	1.8733E-01				1w
	Cerebellum	2.7469E-01				1w
Fig 5C		*Pank1a*	*Coasy*			
	WT-veh vs. KO-veh	2.8964E-03	9.5749E-05			1w
	WT-veh vs. KO-Beza	8.7788E-04	2.1066E-03			1w
	WT-veh vs. KO-Gem	2.0877E-02	1.3012E-03			1w
Fig 6A	WT-veh vs. KO-veh	2.7497E-05				1w
	KO-veh vs. KO-pPanSH	1.6837E-03				1w
Fig 6B		*Coasy*	*Tfrc*	*Ireb2*	*Drd1*	
	WT-veh vs. KO-veh	2.7497E-05	2.2904E-05	3.1837E-02	1.4354E-04	1w
	WT-veh vs. WT-20	1.3324E-03				1w
	WT-veh vs. KO-20	2.0024E-03				1w
	WT-veh vs. KO-8.2				6.1119E-03	1w
Fig 6C	CTL-0 vs. PKAN-0	4.3592E-02				1w
	CTL-0 vs. PKAN-400	3.6906E-02				1w
Fig 6D	within vehicle	1.1068E-05				2w
	within PKAN	1.1001E-03				
Fig 7A		*Coasy*	*Tfrc*	*Drd1*		
	WT-veh-D0 vs. KO-veh-D0	2.1540E-07	1.4228E-06	2.0254E-04		1w
	WT-veh-D0 vs. KO-pPanSH-D7	2.4302E-03	8.9396E-03	1.5690E-02		1w

1w; One-way ANOVA, 2w; Two-way ANOVA analysis

Appendix Figure S1

Aa

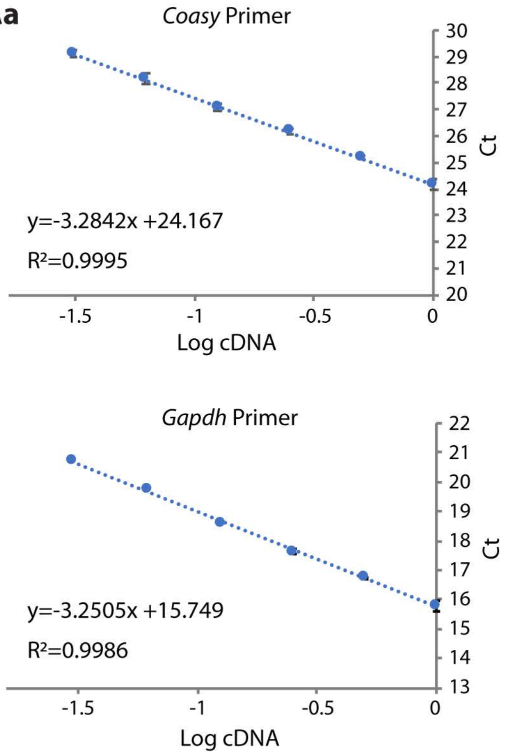

Primer Efficiency

Target	R^2 value	PCR efficiency (%)
Coasy	0.9995	101.6
Drd1	0.9951	110.7
Gapdh	0.9986	103.1
Ireb2	0.9991	101.0
Pank1α	0.9961	100.3
Ppcs	0.9989	104.1
Tfrc	0.9976	102.8

Ab

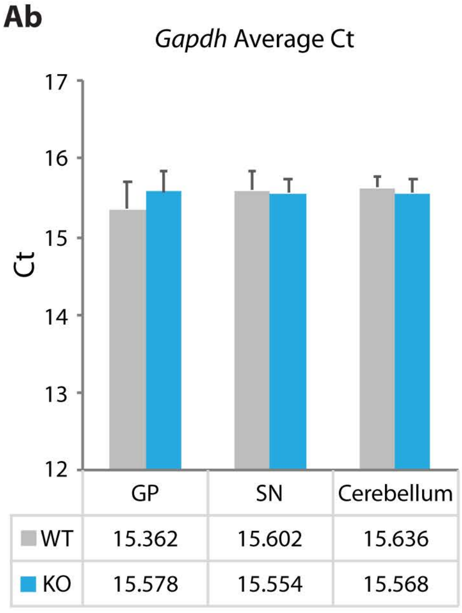

B

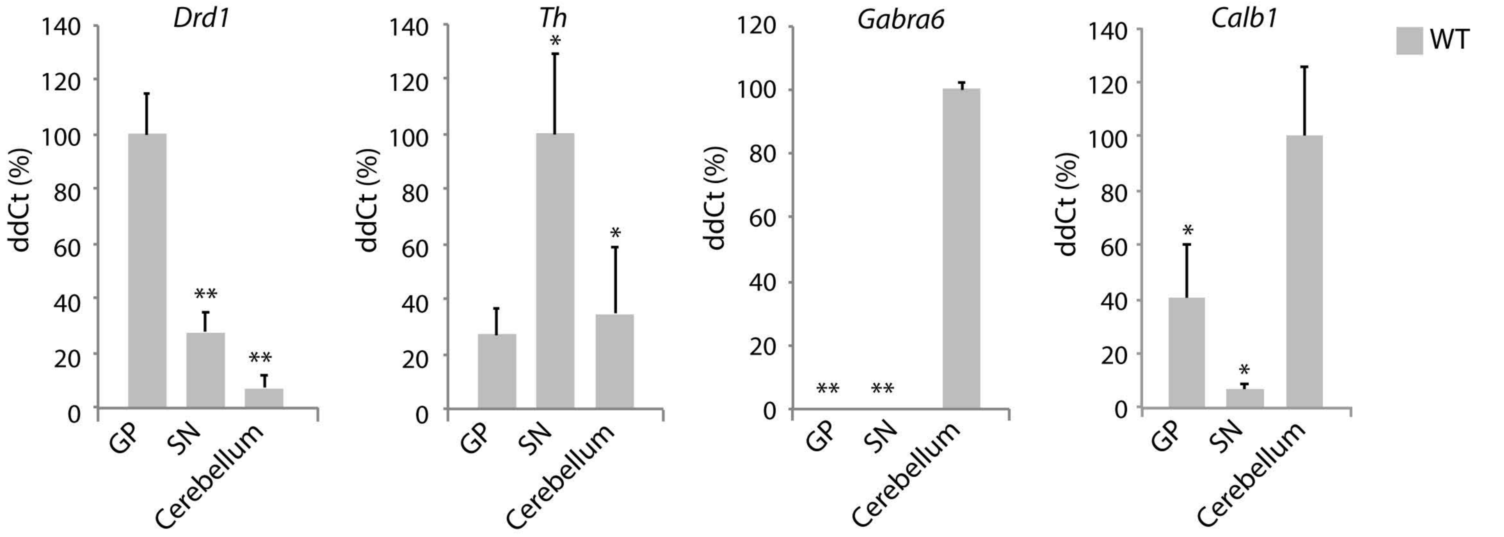

(Aa) Efficiency of primers per target gene showed all primers fit between 90~110% range. QPCR reaction was performed using serially diluted duplicated samples and PCR efficiency was calculated using Efficiency (%) = {-1 +10^(-1/slope)}*100. **(Ab)** Average *Gapdh* Ct showed no difference between genotype nor brain area. **(B)** Genes that are regionally expressed based on public data were selected and quantified by region. Data were evaluated by one-way ANOVA. *P<0.05; **P<0.01. n=3. All graphs represent mean ± s.e.m.

Appendix Figure S2

A

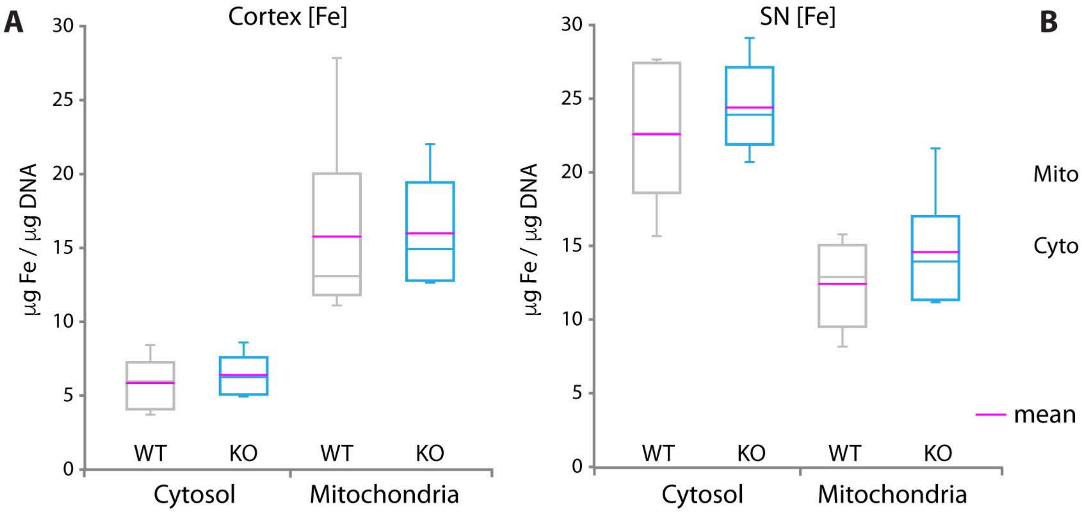

B

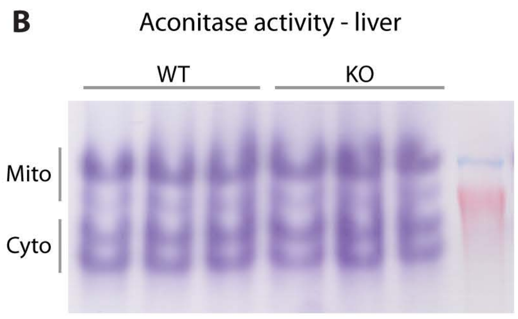

C

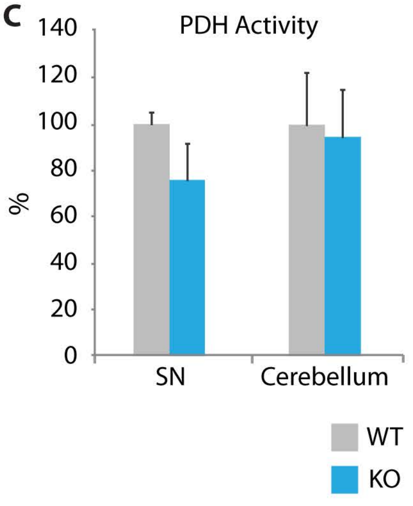

D Two Way ANOVA; PDH Activity in GP

Factor	Comparison	P
Genotype	WT vs. KO	0.011
Drug	pPanSH vs. vehicle	0.054
Drug within WT	vehicle vs. pPanSH	0.540
Drug within KO	pPanSH vs. vehicle	0.002
Genotype within vehicle	WT vs. KO	0.003
Genotype within pPanSH	KO vs. WT	0.951

E Two Way ANOVA; Complex I Activity in GP

Factor	Comparison	P
Genotype	WT vs. KO	0.183
Drug	pPanSH vs. vehicle	0.215
Drug within WT	vehicle vs. pPanSH	0.348
Drug within KO	pPanSH vs. vehicle	0.020
Genotype within vehicle	WT vs. KO	0.018
Genotype within pPanSH	KO vs. WT	0.386

F

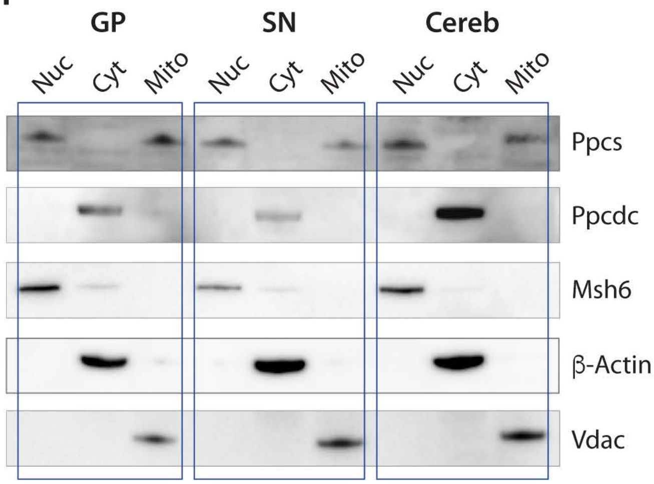

(A) Total iron quantity in cytosol and mitochondria from cortex and SN. The data distribution is presented using a box plot, including minimum, first quartile, median, third quartile, and maximum. The magenta-colored line represents mean value. n=6. **(B)** In-gel aconitase activity using liver lysates from WT and *Pank2* KO mice n=3. **(C)** Activity of pyruvate dehydrogenase in SN and cerebellum by genotype. n=4 for WT, n=5 for KO. All graphs represent mean ± s.e.m. **(D)** Statistical analyses of PDH data presented in Fig. 2D. **(E)** Statistical analyses of complex I data presented in Fig. 2E. **(F)** Subcellular fraction Western blot analyses for Ppcs & Ppcdc in wildtype mouse brain. Msh6, β-Actin, and Vdac were used as subcelluar markers (nuclear, cytosolic, and mitochondrial). Nuc; nuclear, Cyt; cytosolic, Mito; mitochondrial. n=3.

Appendix Figure S3

A

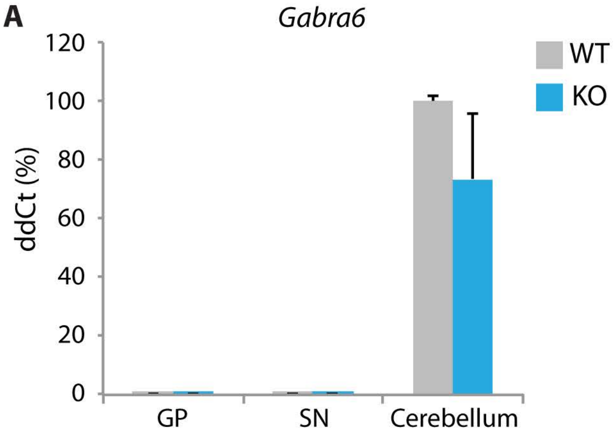

(A) Relative expression of a GABA receptor gene, *Gabra6* in GP, SN, and cerebellum. n=11,4,4 for WT and n=8,4,4 for KO (GP, SN, Cerebellum, respectively). All graphs represent mean ± s.e.m.

Appendix Figure S4

A

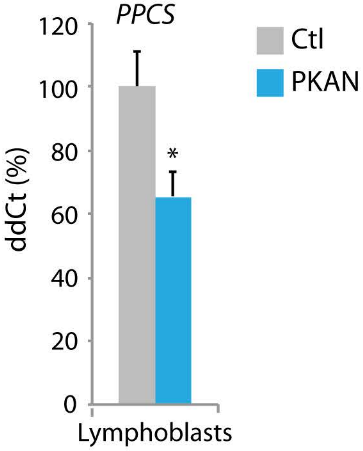

(A) Relative quantification of *PPCS* mRNA from cultured human lymphoblasts by genotype. n=5 for both genotypes. Data were evaluated by one-way ANOVA. *P<0.05. All graphs represent mean ± s.e.m.

Appendix Figure S5

A

One Way ANOVA; *Pank1α*

Comparison	P
WT veh vs. KO veh	0.003
WT veh vs. KO Beza	0.0009
WT veh vs. KO Gem	0.021
KO veh vs. KO Beza	0.346
KO veh vs. KO Gem	0.662
KO Gem vs. KO Beza	0.320

One Way ANOVA; *Coasy*

Comparison	P
WT veh vs. KO veh	<0.0001
WT veh vs. KO Beza	0.002
WT veh vs. KO Gem	0.001
KO veh vs. KO Beza	0.730
KO veh vs. KO Gem	0.607
KO Gem vs. KO Beza	0.684

B

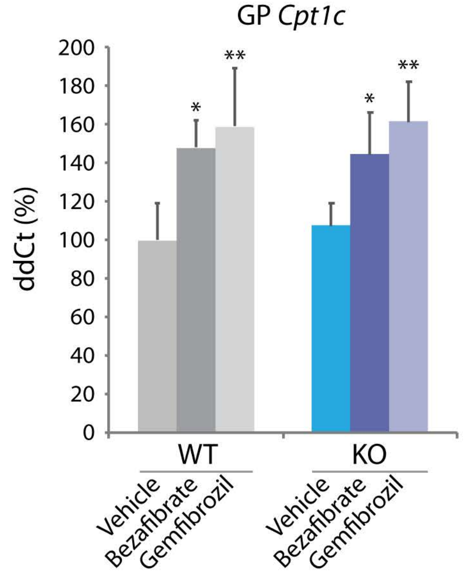

Two Way ANOVA; *Cpt1c*

Factor	Comparison	P
Genotype within vehicle	WT vs. KO	0.522
Drug within WT	vehicle vs. bezafibrate	0.001
Drug within WT	vehicle vs. gemfibrozil	<0.001
Drug within WT	beza vs. gem	0.695
Drug within KO	vehicle vs. bezafibrate	0.021
Drug within KO	vehicle vs. gemfibrozi	<0.001
Drug within KO	beza vs. gem	0.38

(A) Tables of two-way ANOVA results are presented for *Pank1α* and *Coasy* are shown for data presented in Figure 5C. **(B)** Relative quantification of *Cpt1c* mRNA in GP by genotype and treatment status. n=8,8,4 for both genotypes (GP, SN, Cerebellum, respectively). A table of two-way ANOVA results for *Cpt1c* is shown. *P<0.05; **P<0.01. All graphs represent mean ± s.e.m.

Appendix Figure S6

A

One Way ANOVA; *Coasy*

Comparison	*P*
WT veh vs. KO veh	<0.0001
WT veh vs. KO B5	0.0002
WT veh vs. KO PTT	0.003
WT veh vs. KO CoA	0.0001
KO pPanSH vs. KO veh	0.002
KO pPanSH vs. KO B5	0.009
KO pPanSH vs. KO PTT	0.011
KO pPanSH vs. KO CoA	0.033
WT veh vs. KO pPanSH	0.278
KO CoA vs. KO veh	0.963
KO CoA vs. KO B5	0.984
KO CoA vs. KO PTT	0.982
KO PTT vs. KO veh	0.986
KO B5 vs. KO veh	0.976
KO PTT vs. KO B5	0.919

B

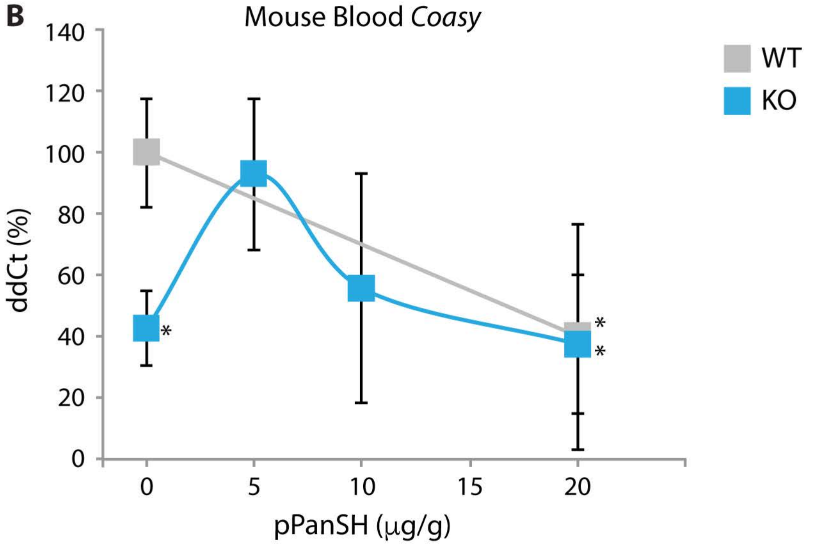

C

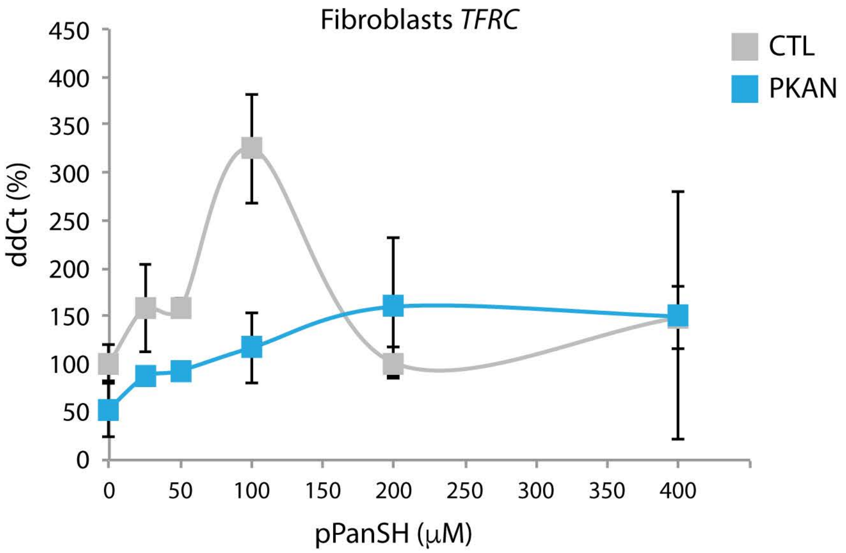

(A) A table of one-way ANOVA results for *Coasy* is shown for data presented in Figure 6A. **(B)** Relative quantification of *Coasy* mRNA from fresh mouse lymphocytes by genotype. n=8 (WT-veh), 5 (KO-veh), 4 (WT-5), 5 (KO-5), 5 (KO-10). 5 (WT-20), 5 (KO-20). *P<0.05. **(C)** Relative quantification of *TFRC* mRNA from cultured human fibroblasts treated with a range of doses of 4′-phosphophopantetheine by genotype. n=3 for both genotypes. All graphs represent mean ± s.e.m.
